# High levels of a comprehensive set of matrix metalloproteinases in endometriotic lesions: validating the key role of cellular senescence in endometriosis pathogenesis

**DOI:** 10.31744/einstein_journal/2025AO1345

**Published:** 2025-07-30

**Authors:** Laura Palmieri, Helena Malvezzi, Bruna Cestari de Azevedo, Eduardo Varejão Díaz Placencia, Eliane Antonioli, Sérgio Podgaec

**Affiliations:** 1 Hospital Israelita Albert Einstein Faculdade Israelita de Ciências da Saúde Albert Einstein São Paulo SP Brazil Academic, Faculdade Israelita de Ciências da Saúde Albert Einstein, Hospital Israelita Albert Einstein, São Paulo, SP, Brazil.; 2 Hospital Israelita Albert Einstein Instituto Israelita de Ensino e Pesquisa Albert Einstein São Paulo SP Brazil Researcher, Instituto Israelita de Ensino e Pesquisa Albert Einstein, Hospital Israelita Albert Einstein, São Paulo, SP, Brazil.; 3 Hospital Israelita Albert Einstein São Paulo SP Brazil Hospital Israelita Albert Einstein, São Paulo, SP, Brazil.; 4 Faculdade Israelita de Ciências da Saúde Albert Einstein Hospital Israelita Albert Einstein São Paulo SP Brazil Postgraduate Program, Faculdade Israelita de Ciências da Saúde Albert Einstein, Hospital Israelita Albert Einstein, São Paulo, SP, Brazil.

**Keywords:** Endometriosis, Cellular senescence, Inflammation, Extracellular matrix, Metalloproteinases, Senescence-associated secretory phenotype

## Abstract

**Objective:**

To investigate the roles of cellular senescence and dysfunctional extracellular matrix metalloproteinases (MMPs) in perpetuating chronic inflammation and facilitating the establishment of endometriotic lesions. By analyzing the MMP activity in the endometrial tissue and peritoneal fluid, we aimed to obtain novel insights into the molecular mechanisms underlying endometriosis-related pathophysiology.

**Methods:**

The endometrial tissue and peritoneal fluid samples were collected laparoscopically from 12 women with endometriosis and 16 healthy controls. Gelatin zymography was performed to assess the activity of MMP-2, and multiplex assays were performed to determine the concentrations of MMP-1 and MMP-3 proteins. Statistical analyses were performed using Generalized Linear Models (GzLM) and SPSS software.

**Results:**

Gelatin zymography revealed higher pro-MMP-2 activity in endometriotic lesions than in eutopic and control endometrium. However, no differences were observed in peritoneal fluid samples. Additionally, MMP-1 and MMP-3 protein levels were elevated in endometriotic lesions compared with those in the eutopic endometrium, whereas only MMP-3 was increased compared with that in the control. No statistical significance was observed for MMP-2, MMP-1, and MMP-3 in the peritoneal fluid samples.

**Conclusion:**

Increased levels of MMP-1, MMP-2, and MMP-3 in endometriotic lesions indicate that endometriosis may have a unique metabolomic signature linked to cell cycle arrest and inflammation. This may contribute to the pathogenesis of endometriosis by facilitating implantation of ectopic endometrium-like tissue in a disturbed immune environment.

## In Brief

This study reveals elevated levels of MMP-1, MMP-2, and MMP-3 in endometriotic lesions, potentially reflecting a senescenceassociated secretory phenotype. No corresponding increase was observed in peritoneal fluid, possibly due to technical or sample-related limitations. These findings support the potential of MMPs as biomarkers and therapeutic targets in endometriosis.

## Highlights

■ MMP-1, MMP-2, and MMP-3 are upregulated in endometriotic lesions.
■ The study findings suggest a senescence-associted secretory phenotype in endometriosis.
■ The study findings support targeting senescence pathways in endometriosis.

## Graphical abstract

**Figure:**
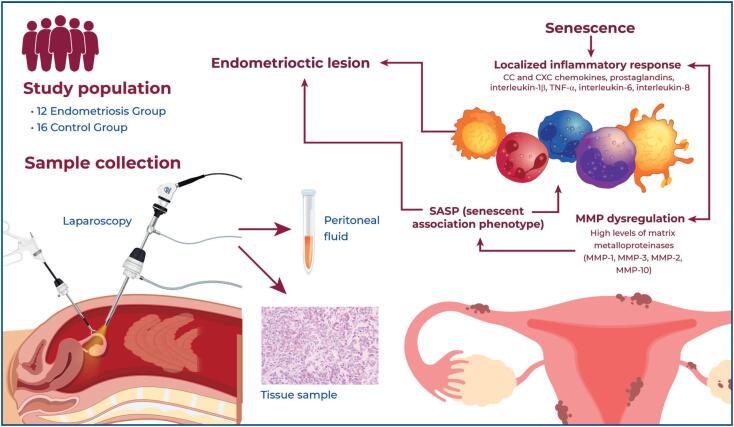

## INTRODUCTION

Endometriosis is a gynecologic condition characterized by the implantation of endometrium-like tissue outside the uterus that typically occurs in women during their reproductive years.^(1)^ It is a benign clinical disease driven by chronic inflammation and is estrogen dependent. Although pelvic pain and infertility are the most common symptoms, the disease is characterized by a wide range of clinical presentations.^(2)^ Treatment includes surgical excision of the lesions and hormonal medications.^(3)^ Despite progress in noninvasive diagnostic approaches and novel treatment paradigms in the field of endometriosis, diagnostic delays challenge disease management, thereby prolonging the time to symptom resolution, which is costly and cumbersome.^(4-6)^

The exact mechanism underlying the pathophysiology of endometriosis is not fully understood. The proposed theories fail to comprehensively explain the disease course and the heterogeneity of its clinical behavior.^(7-9)^ However, sufficient evidence exists highlighting the multifactorial nature of endometriosis, driven by a complex interplay of disrupted hormonal, immune, and inflammatory functions.^(10)^ The development of endometriosis involves resistance to apoptosis, angiogenesis impairment, increased oxidative stress, and dysregulation of the immune system. These processes are intricately linked to the upregulation of proinflammatory factors, growth factors, cytokines, immune cells, and reactive oxygen species (ROS) within the endometriotic lesion microenvironment.^(11,12)^

Despite being a benign disease, endometriosis exhibits malignant-like behavior owing to its ability to adhere to mesothelial surfaces at ectopic sites, degrade extracellular matrix (ECM) components, and invade the surrounding tissues.^(13)^ This phenomenon is partly attributed to the impaired activity of matrix metalloproteinases (MMPs) in the endometriotic lesions. Matrix metalloproteinases are a family of calcium-dependent zinc enzymes responsible for maintaining ECM homeostasis under normal biological conditions.^(14)^ The inflammatory milieu that drives endometriosis has been associated with the overexpression of MMPs in the peritoneal fluid and ectopic stromal cells of affected women, influencing the MMP activity and enhancing the invasive potential of the disease.^(15,16)^ Notably, elevated levels of cytokines, such interleukin-1 beta (IL-1β), tumor necrosis factor alpha (TNF-α), and transforming growth factor β (TGF-β), have been linked to increased transcription and protein secretion of MMP-1 and MMP-3 in patients with endometriosis.^(17)^ Additionally, interleukin-2 (IL-2) and interleukin-27 (IL-27) promote the expression of MMPs by modulating the balance of interferon gamma (INF-γ) and interleukin-10 (IL-10) in ectopic endometrial stromal cells.^(18)^

The inflammatory insults observed in women with endometriosis disrupt the surrounding microenvironment and may induce cell cycle arrest, also known as cellular senescence. Cellular senescence is defined as a prolonged and typically irreversible halt in the cell cycle in response to deleterious stimuli,^(19)^ such as ROS, DNA damage, telomere shortening, mitochondrial degradation, and chromatin disruption.^(20)^ Once cells lose their proliferative capacity, they undergo significant transcriptional changes that alter the expression and secretion of proteins. This ultimately results in the acquisition of a senescence-associated secretory phenotype (SASP).^(21,22)^ Senescence-associated secretory phenotype mediates most of the physiological effects of senescent cells by dynamically perpetuating a chronic inflammatory state.^(23)^ Although the specific secretory profile of the SASP varies with the cell type, it commonly includes increased production of proinflammatory interleukins, such as IL-6, CXCL8 (IL-8), and IL-1, and enzymes involved in ECM remodeling (MMP-1, MMP-2, MMP-3, and MMP-10), serine/cysteine inhibitors (SERPINS), and tissue inhibitors of metalloproteinases (TIMPs).^(24)^

Currently, no molecular marker with absolute specificity for senescence is available. Recently, our research group demonstrated the presence of senescence markers in endometriotic lesions, highlighting the potential relationship between cellular senescence and endometriosis progression and maintenance. In particular, we observed consistently altered expression of senescence-associated proteins, including increased p16^INK4a^, decreased lamin B1, elevated enzymatic activity of senescence-associated β-galactosidase (SA-β-gal), and changes in the secretion profiles of cytokines (IL-4, IL-10, IL-15, and IL-17) as well as oxidative stress (carbonyl, Forkhead Box [FOX], and glutathione [GSH], and oxidized glutathione [GSSG]) in both tissue and peritoneal fluid of patients with endometriosis.^(25-27)^ Moreover, we demonstrated that stimulation of cell cultures with peritoneal fluid from women with endometriosis decreased the expression of IL-2, IFN-γ, IL-17A, and TNF-α while increasing the production of IL-4 and IL-10.^(26)^

Considering the reciprocal inflammatory landscape of the peritoneal microenvironment in endometriosis and the metabolic secretion patterns of SASP, we hypothesized that senescence plays a role in disease progression and maintenance. Chronic inflammation and cellular senescence contribute to the dysregulation of MMPs, which drives tissue remodeling and establishment of ectopic endometrial lesions, directly influencing the pathogenesis of endometriosis. In this abovementioned context, we aimed to evaluate the levels of MMP-1, MMP-2, and MMP-3 in the peritoneal fluid, endometriotic lesions, and eutopic endometrium of women with endometriosis, to obtain new perspectives on the disease mechanism and identify potential avenues for innovative management strategies.

## OBJECTIVE

To investigate the roles of cellular senescence and dysregulated matrix metalloproteinases activity in driving chronic inflammation and endometriotic lesion development, to obtain novel insights into the pathophysiology of endometriosis.

## Methods

### Patients and samples

The study population was a subcohort of the population enrolled for our previous study^(25)^ on the Women's Health Program of *Hospital Israelita Albert Einstein* (HIAE), comprising 12 women with endometriosis (Endometriosis) and 16 control patients without the disease (Control). Samples from women undergoing laparoscopy were collected intraoperatively by a single gynecologic surgeon following the HIAE Institutional Review Board approved protocol (CAAE: 56229916.9.0000.0071; # 5.327.709). Written informed consent was obtained from all participants. The baseline demographic characteristics of participants in both groups were as follows: age between 18 and 50 years; hormonal therapy naïve (*i.e.*, hormonal replacement and combined oral contraceptive) or discontinued at least three months prior to surgery; eumenorrheic menstrual cycles (ranging from 26 to 34 days); non-smoker; and absence of comorbidities (*i.e*., diabetes *mellitus*, cardiovascular disease, and endometrial polyps).

Retrocervical deep-infiltrating endometriotic lesion tissues from 12 women with a histological diagnosis of endometriosis were collected, along with their eutopic endometrium, for the Endometriosis Group. The eutopic endometrium is the tissue lining the uterine cavity that is in its normal position. For the Control Group, 16 endometrium samples were collected from women undergoing laparoscopy for conditions other than endometriosis after macroscopic visualization confirming the absence of endometriotic foci. Samples were stored at −80º C until further analysis. Peritoneal fluid samples were collected and paired with the tissue samples. Endometrial cancer tissue from our tissue consortium was used as a positive control.

### Protein extraction

For total protein isolation, the cryopreserved tissue was ground using ceramic beads, whereas peritoneal fluid samples were homogenized using a Polytron, without protease inhibitors. The lysates were homogenized twice by sonication. The supernatants were recovered, and protein concentrations were determined using the Pierce™ BCA Protein Assay kit (Cat No. 23225, Thermo Fisher™, Waltham, MA, USA), according to the manufacturer's instructions. To optimize the densitometric analysis and prevent overexposure, the protein extracts were diluted. Tissue and peritoneal fluid samples were diluted 2- and 6-fold, respectively.

### Gelatin zy mography

Gelatin zymography was performed to analyze the enzymatic activity of MMPs. The resulting gels were scanned and subjected to densitometry to quantify the intensity of the bands corresponding to the MMP activity. Equal amounts of total protein (20*µ*g) from tissue and peritoneal fluid samples were loaded on a 10% SDS-polyacrylamide gel containing 0.2% gelatin (Sigma-Aldrich, München, Germany) to evaluate the MMP-2 activity. Endometrial cancer tissue homogenate was used as a positive control, and 10x phosphate-buffered Saline (PBS) was used as a negative control. For electrophoresis, 20*µ*g protein made to a final volume of 16*µ*L with PBS was loaded in each well along with 4*µ*L of nonreducing sample buffer (4% sodium dodecyl sulfate [SDS]; 0.125 M Tris-HCl [pH 6.8], 20% glycerol and 0.001% bromophenol blue). After electrophoresis, gels were washed three times with 2.5% Triton X-100 for 15 min and incubated for 36 h at 37ºC in developing buffer (50mM Tris-HCl, pH 7.4; 10mM CaCl_2_; 0.15 M NaCl) on an orbital shaker. The gels were stained with 0.05mg/mL Coomassie Brilliant Blue G-250 (Bio-Rad^®^, Hercules, California, USA) and destained for 1 h with 8% acetic acid and 4% methanol. Each assay was performed at least twice. Images were captured using the iBright^®^ Analysis Software photodocumentator (Thermo Fisher Scientific, Waltham, MA, USA). Proteolytic activity was measured by quantifying clear zones against a dark blue background, indicating the lysis of gelatin, using the ImageJ Software. Proteinase activity was expressed as units per milligram of protein, and the size of the lysed area and intensity were proportional to the protein content.

### Multiplex assay

A multiplex protein assay was performed using tissue and peritoneal fluid homogenates for MMP-1 and MMP-3 profiling of the samples. MMP-2 was not analyzed in the multiplex protein assay because the multiplex kit used in this study did not include MMP-2 among its detectable targets within the analyte panel predetermined by the manufacturer. The analysis was performed using the xMAP technology (Luminex, Austin, TX, USA) for multiplexed quantification based on magnetic beads. Ten markers, including MMP-1 and MMP-3, were simultaneously quantified in samples using the Human MMP Premixed Magnetic Luminex^®^ Performance Assay (Cat No. FCSTM07-03; R&D Systems, Minneapolis, MN, USA) following the manufacturer's instructions. The samples were 5-fold diluted using the dilution buffer included in the assay kit. Data were collected using the xPonent software (Luminex).

### Statistical analysis

All data analyses were performed using the SPSS Statistics v. 26 software. Results are expressed as mean±standard deviation of the mean and are displayed as bar graphs. For group comparisons, we used the Generalized Linear Model (GzLM) because this test does not require a normal distribution of errors or homoscedasticity,^(28)^ considering the limited sample size in this study. The distribution choice was based upon the Akaike Information Criterion (AIC) available in the tool. Results with a p<0.05 were considered statistically significant.

## RESULTS

Patient characteristics are summarized in table 1. The mean age in the Control Group was 39 years (95%CI, 34-43) and the mean body mass index (BMI) was 29.09 (95%CI= 26.08-32.09). The Endometriosis Group had a slightly lower mean age of 36 years (95%CI, 33-40) and a lower mean BMI of 24.55 (95%CI, 21.78-27.31). Most patients in both the groups (87.5% in the Control Group and 67% oin the Endometriosis Group) were in the proliferative endometrial cycle phase.

**Table 1:** Patient characteristics: age, body mass index, and endometrial menstrual cycle phase

Variables	Control Group (n=16)	Endometriosis Group (n=12)
Age (Years)	39 [34–43]	36 [33–40]
BMI	29.09 [26.08–32.09]	24.55 [21.78–27.31]
Proliferative endometrial cycle phase	14 (87.5%)	8 (67%)

Age and BMI values are presented as means and 95% confidence interval ranges. Endometrial menstrual cycle data are presented as absolute values and percentages.

BMI: body mass index.

**Figure 1:**
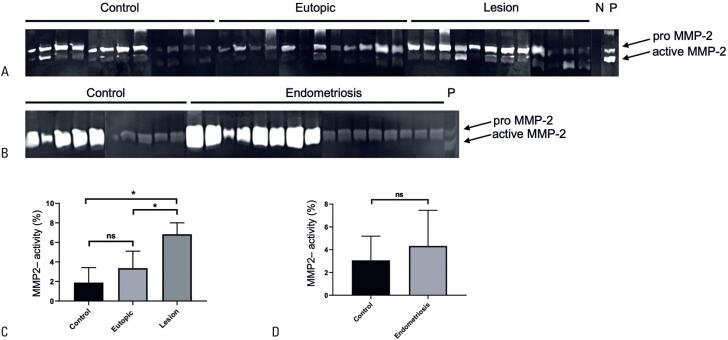
Representative gelatin zymograms of total protein lysates showing (A) tissue samples from the Control Group (healthy endometrium only) and from the Endometriosis Group (eutopic endometrium and ectopic endometriotic lesions), and (B) peritoneal fluid samples from Control and Endometriosis Groups. Arrows indicate the pro- (72 kDa) and active (62 kDa) forms of MMP-2. P, positive control; N, negative control. Densitometric quantification of MMP-2 levels in (C) tissue samples and (D) peritoneal fluid samples

Total MMP-2 activity was analyzed using gelatin zymography of tissue biopsy and peritoneal fluid lysates from women with and without endometriosis. Based on the protein size, we could distinguish the latent pro- and active forms of MMP-2 (∼72kDa and ∼62kDa, respectively) in all the analyzed samples. The areas of enzymatic activity appeared as clear bands, demonstrating the proteolytic digestion of gelatin in the polyacrylamide gel, such that the band intensity was proportional to protein content (Figure 1A and 1B). Most MMPs are synthesized as proenzymes and secreted prior to their conversion to an active form.^(29,30)^ The majority of endometriotic lesions showed increased gelatinolytic bands for the proenzymatic form of MMP-2 compared with either eutopic or control endometrium (Figure 1A). Moreover, a distinguishable proteolytic digestion pattern was not observed in peritoneal fluid samples from the Control and Endometriosis Groups (Figure 1B).

*p<0.05.

Data are normalized to the group mean and expressed as percentages.

MMP: matrix metalloproteinase; NS: not significant.

Quantitative analysis of the relative activity of pro-MMP-2 via densitometry confirmed these observations. Densitometric data were normalized against the average values and presented as percentages. The average activity of MMP-2 was significantly increased in the endometriotic lesions compared with that in the eutopic endometrium (6.8%±1.2 *versus* 1.9%±1.5; p<0.01) and control endometrium (6.8%±1.2 *versus* 3.4%±1.7; p<0.01). No statistically significant difference was observed between the control and eutopic endometrium (Figure 1C). In the peritoneal fluid samples, no statistically significant difference was observed between the Endometriosis and Control Groups in terms of pro-MMP-2 activity levels as evident from the results of densitometric analysis (Figure 1D).

**Figure 2:**
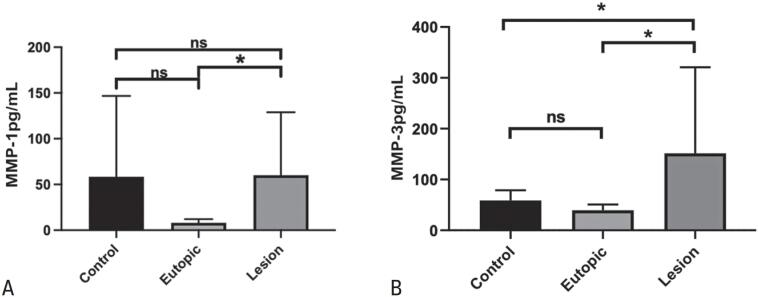
Activity of MMP-1 and MMP-3 in endometriotic lesions, as measured in tissue and peritoneal fluid samples using multiplexed bead-based ELISA (Luminex xMAP technology). (A) MMP-1 levels were significantly higher in endometriotic lesions compared to eutopic endometrium from (p<0.05), with no significant difference observed between endometriotic lesions and control endometrium. (B) MMP-3 activity levels were significantly increased in endometriotic lesions compared to both eutopic endometrium and control endometrium (p<0.05)

The association of MMP-1 and MMP-3 with the SASP and endometriosis pathophysiology prompted us to investigate the levels of these ECM components in our samples using the Luminex xMAP technology. All analytes were detected in the tissue and peritoneal fluid samples. The MMP-1 levels were higher in endometriotic lesions than in the eutopic endometrium (68.41±71pg/mL *versus* 7.99±3.76pg/mL, p<0,05). However, similar findings were not observed for the comparison with the control endometrium (68.41±71pg/mL *versus* 58.44±88.23pg/mL, p>0,05). The protein levels of MMP-3 in endometriotic lesions were higher than in the eutopic endometrium (151±169pg/mL *versus* 39.4±11,4pg/mL, p<0.05) and control endometrium (151±169pg/mL *versus* 59.0±20pg/mL, p<0.05) (Figure 2). Our data indicate a close association between MMPs and the endometriotic lesion microenvironment, suggesting a potential link to a senescent cell metabolomic state.

*p<0.05.

Bars represent mean ± standard deviation. MMP levels are expressed in pg/mL.

MMP: matrix metalloproteinase; NS: not statistically significant.

## DISCUSSION

In this study, we determined the levels of a comprehensive set of MMPs, previously described as senescence biomarkers,^(21,31)^ to explore the potential relationship between the pathogenesis of endometriosis and cellular senescence. The increased levels of MMP-2 and MMP-3 in endometriotic lesions compared with those in the eutopic and control endometrium could be a distinct metabolomic signature in endometriosis, potentially resulting from cell cycle arrest. Moreover, the unique secretion pattern of MMP-1 in endometriotic lesions and eutopic endometrium highlights behavioral changes within the endometrium-like tissue that is absent in a non-disturbed intrauterine microenvironment. The lack of significant changes in the peritoneal fluid among the analyzed groups may be attributable to an excessive number of proteins rather than those of interest. This may have compromised the accuracy of the densitometric evaluation of these samples, as indicated by overexposed bands (Figure 1B). Additionally, baseline demographic data (Table 1) showed that the Control Group presented with an advanced age, had a higher BMI, and predominantly consisted of individuals in the endometrial proliferative phase. This strengthens our analysis as the nonstatistical trends indicate that the Endometriosis Group was less affected by common risk factors for disrupted MMP activity, which could ultimately be associated with senescence.

Endometriosis remains a clinically challenging disease, marked by a delayed interval between symptom onset and diagnosis, which prolongs the duration of pain and severely affects the quality of life of women.^(32)^ Clinical examination is insufficient to differentiate the disease from other chronic pelvic complaints, despite advances in noninvasive imaging tools, all of which require skilled specialists who are in short supply. Laparoscopic visualization of endometriotic foci remains the standard diagnostic and therapeutic option for women for whom symptom severity justifies the risks of the procedure.^(33)^ However, a substantial proportion of women continue to be symptomatic even after the excision of macroscopic lesions, and prospective therapeutic options with curative intent are lacking.^(34)^ Identification of precise molecular phenotypes in endometriosis is urgently needed to provide functional insights into the pathogenesis of the disease and to guide the development of targeted and more effective therapies.

After surpassing host immune defenses, the endometrium-like tissue must adhere to and proliferate at the ectopic site. Regardless of the origin of the endometriotic lesion (*i.e*., coelomic metaplasia, tubal reflux, and lymphatic and vascular metastasis)^(7,9,35)^ cells must invade the surrounding tissue and develop an optimal microenvironment to persist within the pelvic compartment. The success of ectopic implantation depends on the ability of the implant to interact with the ECM of the host tissue. MMPs facilitate these processes by enabling proteolytic digestion and remodeling of the ECM and non-matrix components.^(15)^

MMPs are calcium-dependent zinc enzymes that degrade ECM elements. They are classified by their substrate specificity as collagenases (MMP-1, −8, and −13), gelatinases (MMP-2 and −9), and stromelysins (MMP-3 and −10), which degrade collagen, gelatin, and proteoglycans, respectively. MMPs are critical for ECM remodeling, which is essential for tissue invasion, neovascularization, and the progression of diseases involving malignant transformation, tumor progression, age-related pathologies, and cellular senescence.^(36)^ Previous studies have shown that the MMP activity is disrupted in endometriosis.^(37)^ Increased levels of MMPs, such as MMP-2, MMP-9,^(38,39)^ MMP-3,^(40)^ have been observed in the endometriotic tissue and serum of women with endometriosis, indicating their potential role in disease progression. Using three-dimensional culture models mimicking the architecture of endometriosis lesions, Stejskalová et al. demonstrated that stromal cell lines could migrate and establish ectopic lesions via collagen type I remodeling. This process was significantly suppressed in the presence of MMP inhibitors (TIMPs).^(41)^ Matrix metalloproteinases expression is also influenced by hormonal fluctuations during the menstrual cycle in an estrogen-dependent manner, which is relevant to the development of endometriosis.^(42)^ Therefore, the elevated levels of MMPs in endometriotic lesions compared with those in the eutopic endometrium and Control Group in this study indicate that these enzymes could be pivotal in driving chronic inflammation and tissue remodeling, which are key processes underlying the pathogenesis of endometriosis.

Matrix metalloproteinases play critical roles in cellular senescence.^(21)^ Senescent cells exhibit altered MMP activity and changes in the secretome pattern, including increased expression of inflammatory cytokines, such as IL-1, IL-6, IL-8, CCL-8, CCL-13, and CCL-2, growth factors (IGF and CSFs), and other soluble molecules (PE2).^(43)^ Senescence is often characterized by high levels of MMPs (MMP-1, MMP-3, MMP-2, and MMP-10), which are part of the SASP.^(44,45)^ Studies on human fibroblast cultures subjected to senescence have shown that senescent fibroblasts secrete numerous MMPs, particularly MMP-1, MMP-3, and MMP-10.^(46)^ MMP-2 has also been associated with senescence, participating in various SASP cleavage processes, and its levels were increased under this scenario.^(47)^ No biomarker is available to assess senescent cells with absolute specificity because of the highly heterogeneous nature of the process, which depends on triggering stimuli, tissue and cell type, and other factors. The increased activity of MMPs, especially MMP-1, MMP-2, MMP-3, MMP-9, and MMP-10, is an indirect indicator of cellular senescence, as the disrupted function is known to be associated with SASP.^(48)^

The reciprocal effects of the proinflammatory microenvironment in endometriotic and senescent cells, also characterized by dysfunctional MMP activity, is suggestive of an association between senescence and the pathogenesis of endometriotic lesions. Previous research by our group has consistently demonstrated this reciprocal relationship. Correlation between senescence markers, such as IL-17A, p16^Ink4a^, lamin b1, IL-1α, and IL-2 and endometriosis have been observed.^(25,27)^ Additionally, increased levels of IL-1β and IL-4 were detected in endometriotic lesions compared with that in the eutopic endometrium. IL-1β is a proinflammatory cytokine associated with inflammatory and cell proliferation processes, as well as a precursor of senescent state. Furthermore, consistent oxidative stress (OS) has been found to be associated with the dysregulation of mitogen-activated protein kinase (MAPK) pathways.^(26)^ The MAPK pathway triggers a senescence cascade and upregulates proteins such as p16^Ink4a^, which are responsible for maintaining the growth arrest state indefinitely via the activation of the retinoblastoma (RB) family of proteins. The negative impact of OS on the expression of senescence markers in stromal cells of endometriotic lesions was evidenced by an increase in SA-β-gal activity and upregulation of p16^Ink4a^ along with a subtle decrease in lamin B1 expression. These findings support the role of senescence in the development of endometriosis.^(26)^

To the best of our knowledge, this is the first study to explore the relationship of disrupted MMP levels and cellular senescence with endometriosis. By identifying a prosenescent profile in endometriotic lesions, as evident from elevated MMPs levels, we provide clinical insights for the development of noninvasive and point-of-care diagnostic tools, such as the evaluation of prosenescent biomarkers to diagnose or rule out endometriosis. This could help reduce diagnostic delays, lower costs, and make diagnostic algorithms more accessible in settings with limited access to specialized healthcare. Understanding the pathogenesis of endometriosis would also lead to the identification of novel therapeutic targets that could alter disease progression, meaningfully impacting the management of this burden condition.^(49)^ Promising results from preclinical models and clinical trials suggest that senolytic agents may offer therapeutic options with a curative intent for chronic inflammatory diseases. One such option is dasatinib combined with quercetin (D+Q), a second-generation BCR-ABL1 kinase inhibitor approved by the FDA for the treatment of chronic myeloid leukemia.^(50)^ A phase 1 study involving patients with idiopathic pulmonary fibrosis, a disease recognized for its high lethality associated with senescence, demonstrated that D+Q improved the physical capacity of these patients.^(51)^ This underscores the therapeutic potential of targeting MMPs for the treatment of chronic inflammatory diseases and as a promising surrogate in endometriosis management. Senolytic agents should be further explored to treat benign conditions such as endometriosis, with improved drug tolerability profiles, dose optimization, and targeting of specific pathways to balance therapeutic efficacy and minimize side effects.

The results of the present study should be interpreted with caution. While qualitative assessment of MMPs provides a systematic approach for investigating the presence of known senescence-related biomarkers in endometriotic lesions, the limited sample size and technical reproducibility pose obstacles to extrapolating our findings. Cellular senescence is a dynamic and contextual process with a plethora of molecular signatures and an absence of a single universal marker. Therefore, subsequent analyses with larger prospective sample sizes and a broader panel of senescence-related biomarkers are necessary to confirm our findings. Although presented separately, the experiments were carried out in a subset of subjects from the cohort in our previous study, which reinforces the association between endometriosis and senescence and strengths our findings.^(25,26)^ Moreover, while we acknowledge that variables, such as BMI and the menstrual cycle, could significantly influence MMP expression, the pilot nature of the study limited the scope of our investigation to exploring the overall expression of MMPs across groups. Further studies in larger, well-annotated cohorts are required to investigate these relationships.

Endometriosis is a chronic inflammatory disease that affects millions of women worldwide and has detrimental effects on their social, economic, and well-being. Unraveling the subphenotypes of the disease is urgently needed to advance its management and treatment, ultimately improving clinical outcomes.

## CONCLUSION

The increased levels of MMP-1, MMP-2, and MMP-3 in endometriotic lesions observed in this study indicate that endometriosis may have a distinct metabolomic signature linked to cell cycle arrest and inflammation. The altered matrix metalloproteinases activity may contribute to the pathogenesis of endometriosis by facilitating the implantation and persistence of ectopic endometrium-like tissue in a dysregulated immune environment. Our findings support the potential role of cellular senescence in disease progression and highlight the need for further research on matrix metalloproteinases-targeted diagnostic and therapeutic approaches to improve the management of endometriosis.
